# Effects of Resistance Exercise on Cognitive Performance and Depressive Symptoms in Community-Dwelling Older Chinese Americans: A Pilot Randomized Controlled Trial

**DOI:** 10.3390/bs13030241

**Published:** 2023-03-09

**Authors:** Mei-Lan Chen, Ying-Jung Wu, Mi-Jung Lee, Sung-Lin Hsieh, Ing-Jy Tseng, Li-Sheng Chen, Douglas S. Gardenhire

**Affiliations:** 1 School of Nursing, Byrdine F. Lewis College of Nursing and Health Professions, Georgia State University, Atlanta, GA 30303, USA; 2 Department of Respiratory Therapy, Byrdine F. Lewis College of Nursing and Health Professions, Georgia State University, Atlanta, GA 30303, USA; 3 Department of Computer Science, School of Engineering, Vanderbilt University, Nashville, TN 37235, USA; 4 School of Gerontology and Long-Term Care, Taipei Medical University, Taipei 11031, Taiwan

**Keywords:** resistance exercise, strength exercise, cognitive function, depression, physical activity, older adults, randomized controlled trial, cognition, executive function, memory

## Abstract

Previous literature has suggested physical exercise may improve cognitive impairments and mitigate depressive symptoms. However, few studies examined the impact of resistance exercise intervention on cognition and depression in older Chinese Americans. The purpose of this pilot study was to assess the effects of resistance exercise training on cognitive performance and depressive symptoms among community-dwelling older Chinese Americans. The study was a two-arm randomized controlled trial with pre-test/post-test design. Thirty older adults were randomly assigned into the resistance exercise intervention group or the wait-list control group. Participants’ cognitive performance and depressive symptoms were evaluated at baseline (pre-test) and at 12 weeks (post-test). The results showed that there were significant differences between the intervention and control groups on changes in symptoms of depression, global cognitive function, visuospatial/executive functions, attention, language, and orientation. However, there were no significant differences between both groups on changes in naming, abstraction, and delayed recall domains. The findings of this study suggest that resistance exercise training has a positive impact on improving cognitive performance and depressive symptoms in older adults.

## 1. Introduction

Aging is characterized by the involuntary declines in muscle strength and physical function, which is the primary factor contributing to disability in the elderly [1]. Regular exercise could potentially prevent or delay the onset of age-related disability and functional dependence. Previous research has shown that resistance exercise training significantly improved muscle quality index and physical performance in the older adult population [2]. The current physical activity guideline for Americans recommends that older adults do muscle-strengthening exercise, which involves major muscle groups at least two days per week [3,4]. However, Asian Americans are less likely to engage in resistance exercise than White Americans [5]. There were 28.2% of White Americans, compared to only 26.8% of Asian Americans, that performed muscle-strengthening activities on two or more days per week [5]. In addition, prevalence of resistance exercise among adults decreased significantly as age increased. The percentage of older adults engaging in regular resistance exercise that met the current physical activity guidelines was only 18.6% among those aged 65 years and over [5]. Obviously, promoting resistance exercise for Asian Americans and the elderly is crucial.

Cognition plays a vital role in communication and functional independence [6]. With the aging progress, cognition has been proven to decline due to the density and the volume losses from the frontal, parietal, and temporal cortices [7,8]. Those brain areas are also related to cardiovascular function [8]. Moreover, Alzheimer’s disease, the most common type of dementia, is a progressive brain disorder linked to cognitive function loss [9]. Nearly 6.2 million people aged 65 years and older had Alzheimer’s disease in the United States [10]. The number is expected to keep increasing to 13.8 million by 2060 [10,11]. Alzheimer’s disease and cognitive impairments have a profound burden on older adults and their family caregivers [10,12]. Hence, it is critically important to develop effective interventions for promoting cognitive health among older adults.

Existing systematic reviews and/or meta-analyses suggested that resistance exercise interventions can improve cognitive function in older adults [13,14,15,16,17,18]. Yet, the association between resistance exercise and cognitive performance has not been fully examined in diverse aging populations. Previous studies have shown that resistance training has positive effects on cognition in older adults [7,19,20,21]. However, most studies focused on the relationships between resistance exercise training and global cognitive function. The impact of resistance exercise on specific cognitive domains remains unclear.

Depression is a common mental disorder among older adults [22]. As age progresses, various social and health issues start to become apparent. One of them is falling victim to having chronic diseases, which would lead to a higher chance of developing depression [23]. Depression is also closely linked to geriatric suicide [22]. Elders comprise 12% of the US population but make up nearly 18% of the suicides [24]. Hence, it is imperative to manage and relieve symptoms of depression among older adults. The main treatment for depression involves psychological and pharmacological interventions [25]. However, antidepressant medication has the possibility of increasing the severity of depressive symptoms [26]. Also, the effect of psychotherapy remains inconclusive [27]. Therefore, alternative treatments such as physical activity interventions should be taken into consideration in battling depression. Studies revealed that exercise interventions improved symptoms of depression among older adults [28,29,30]. Previous research also suggested that resistance exercise can decrease depressive symptoms in older adults [31,32,33,34,35]. However, Chin et al. found that resistance exercise did not have significant effects on improving depression among older adults [36]. The association of resistance exercise’s effect with the improvements of depression in older adults is still lacking clarity. In addition, Asian Americans are less likely to report mental health disorders and seek help for mental illness than their counterparts [37,38]. Compared to White Americans, Asian Americans are less likely to receive mental health care [39]. Hence, the development of effective interventions for enhancing Asian Americans’ mental health is essential.

Physical activity interventions have shown to be potentially beneficial for preventing or improving depression and cognitive impairments in older adults. Many studies have emphasized the relationships of aerobic exercise with cognitive performance and depressive symptoms [9,13,20,28,29,30,40]. However, there has been limited research into examining the effects of resistance exercise on depression and cognitive functioning among community-dwelling older adults. Also, previous studies did not focus on the population of Chinese American elders. The effect of resistance exercise on cognition and depression in older Chinese Americans remains unknown. To address the unmet need, this study aimed to examine the effect of a 12-week resistance exercise intervention on cognitive performance and depressive symptoms among community-dwelling older Chinese Americans.

## 2. Materials and Methods

### 2.1. Ethical Approval and Trial Registration 

The study was approved by Georgia State University Institutional Review Board (IRB number: H18308). The randomized controlled trial has been retrospectively registered at ISRCTN Registry (ISRCTN12284883) [41].

### 2.2. Study Design and Randomization

This pilot study was a two-arm randomized controlled trial with a pre-test/post-test design. A convenience sample of community-dwelling older Chinese Americans was recruited from an adult day healthcare center in Georgia, USA. Participants were recruited through recruitment flyers. Individuals with an interest in participating were screened for eligibility. Prior to data collection at baseline, the process of informed consent was completed by participants. After completing the process of informed consent and baseline assessments, participants stratified by gender and age (60–74 vs. 75–89) were randomly assigned into two groups (1:1 ratio): the intervention group (resistance exercise intervention group) or the control group (wait-list control group). The randomization using the random number generator in SPSS was conducted by an individual who was blinded to participant details. In this pilot study, it was impossible to blind the participants and interventionists to the group due to the nature of the resistance exercise intervention. 

### 2.3. Participants

Participation in this study was completely voluntary. Participants had the right to drop out at any time. All participants met the following inclusion criteria: (a) age 60 or older (age 60–89); (b) self-identifying as Chinese American; (c) able to speak Mandarin; (d) able to walk independently without assistive devices; (e) did not engage in any resistance exercise programs during the 6 months prior to this study; (f) ability to follow verbal and visual instructions; (g) ability to give informed consent and complete the assessment battery. 

Exclusion criteria included the following limiting health conditions or medical diagnoses: (a) blood pressure > 160/100 mmHg or severe complications of hypertension, such as aneurysm, heart failure, or metabolic syndrome; (b) a history of coronary artery disease, cardiac surgery, heart attack, or stroke in the past 3 months; (c) health-related problems that would interfere with participation in the exercise program, such as angina, uncontrolled diabetes, or serious cardiac arrhythmias; (d) active treatment for cancer or substance abuse; (e) a history of upper-extremity, lower-extremity, hip, or back surgery in the past 3 months, or (f) severe cognitive impairments, such as signs of psychosis, dementia, not being oriented to time, place, or person.

### 2.4. Intervention

During the study period, the intervention group received 12-week resistance exercise training, while the wait-list control group did not receive the exercise intervention. The wait-list control group was asked to maintain their usual activities. After completing the post-test, the wait-list control group had an opportunity to receive a delayed intervention (the same 12-week resistance exercise training as the intervention group) if desired. This resistance exercise program was based on recommendations for older adults from the National Institute on Aging (NIA), USA [42]. The NIA’s exercise and physical activity guidelines recommend that older adults should do resistance exercise on two or more days a week for 30 min per session [42]. In this study, the exercise intervention comprised upper-extremity and lower-extremity progressive resistance training focusing on all major muscle groups (legs, hips, back, abdomen, chest, shoulders, and arms). The 12-week exercise intervention included 50 min group exercise sessions two times a week. The intensity of exercise was determined based on the Borg Rating of Perceived Exertion (RPE) [43]. The scores of the Borg Scale range from 6 (no exertion at all) to 20 (maximal exertion). The intensity of exercise was gradually increased to a perceived exertion of 13–16 on the 6–20-point Borg Scale. Each exercise session consisted of a 10 min warm-up (e.g., walking), 30 min resistance exercise training (e.g., hand grip, wrist curl, arm curl, arm raise, knee curl, leg raise, toe stand), and a 10 min cool-down (e.g., flexibility exercises) [42,44,45]. A detailed description of warm-up, resistance exercise, and cool-down has already been reported by the NIA [42]. 

Each exercise session was led by a bilingual Mandarin-English speaking registered nurse and a trained graduate research assistant at an adult day healthcare center in Georgia, USA. Participants were closely supervised by researchers. During each exercise session, participants’ blood pressure, heart rate, the intensity of exercise, and level of fatigue were assessed. Blood pressure and heart rate were measured using a digital blood pressure monitor (Omron, HEM-907XL). The Borg Scale was used to assess the intensity of exercise [43]. The level of fatigue was rated on a scale ranging from 0 to 10; higher scores indicated higher levels of fatigue. Rest periods were provided as needed during exercise sessions.

### 2.5. Measurements

#### 2.5.1. Demographic Characteristics

In this study, a self-reported demographic questionnaire was used to obtain participants’ demographic information, health conditions, medical history, and research-related items.

#### 2.5.2. Montreal Cognitive Assessment 

Cognitive performance was measured using the Montreal Cognitive Assessment (MoCA) at baseline and at 12 weeks [46]. The MoCA is a suitable, sensitive, and specific tool to test specific cognitive domains in visuospatial/executive functions, naming, memory, orientation, attention, delayed recall, language, and abstraction in older adults [47]. The Chinese version of MoCA has well-established validity and reliability in older Chinese adults [48,49]. Total scores of the MoCA range from 0 to 30. Higher scores indicate better cognitive performance.

#### 2.5.3. Geriatric Depression Scale 

The 15-item Geriatric Depression Scale (GDS) was used to assess participants’ symptoms of depression at baseline and 12 weeks [50]. The GDS is a sensitive and validated screening tool for self-rating depressive symptoms [51]. It is widely used in older adults for the assessment of depression. The Chinese version of GDS has been validated in older Chinese population with good reliability and validity [52,53]. Total scores of the GDS range from 0 to 15. Higher scores reflect greater depressive symptoms.

### 2.6. Statistical Analysis

Baseline characteristics were analyzed using descriptive statistics. The Kolmogorov–Smirnov test was applied to check the normality of the data. Categorical variables used Chi-square tests, and continuous variables used independent t-tests or Mann–Whitney U tests to compare differences between the intervention and control groups at baseline. The paired t tests or Wilcoxon signed-rank tests were performed to examine differences between the pre-test and post-test on depressive symptoms and cognitive performance for the intervention group and for the control group. The Mann–Whitney U tests were employed to test the difference of change from pre-test to post-test on depressive symptoms and cognitive performance between the intervention and the control groups. Effect sizes were calculated using *r* = Z/sqrt(N) and interpreted as small (*r* = 0.1), medium (*r* = 0.3), and large (*r* = 0.5) [54,55]. The software G*Power 3.1 was used for sample size estimation for future studies. Statistical analyses were performed using Statistical Package for the Social Sciences (SPSS, version 28). A two-sided *p*-value < 0.05 is considered statistically significant.

## 3. Results

### 3.1. Participants Flow 

**Figure 1:**
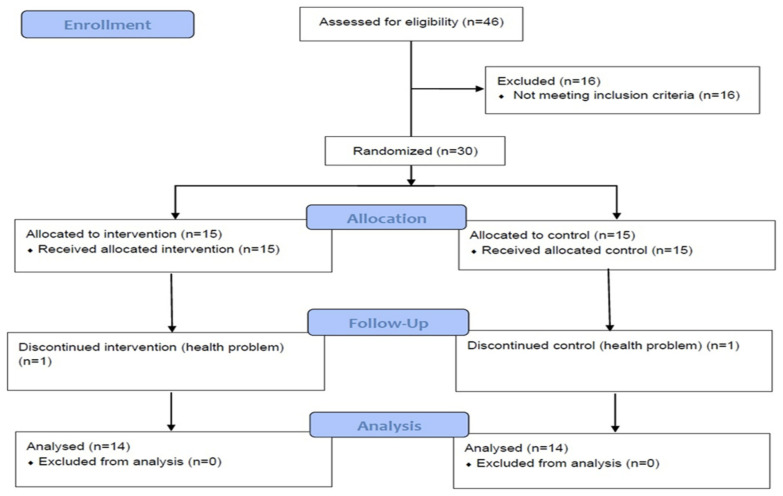
Participants’ flow diagram [56].

Figure 1 shows the trial flow diagram of this study [56]. Thirty participants met eligibility criteria. Of the 30 participants, 15 were randomly assigned to the resistance exercise intervention group, and 15 were randomly assigned to the wait-list control group. Two participants dropped out of the study due to health problems. A total of 28 subjects were included in the analyses.

### 3.2. Characteristics of the Sample

Table 1 indicates baseline characteristics of the participants. The mean age of subjects was 77.68 ± 5.11 years; the majority of participants were women (78.6%). As shown in Table 1, there were no significant differences between the intervention and control groups on sample characteristics at baseline (all *p* ≥ 0.05).

### 3.3. Within-Subjects Comparisons of Pre–Post Changes for Each Group

Table 2 shows the changes from baseline (pre-test) to post-test on depressive symptoms and cognitive performance for the intervention group and for the control group. As shown in Table 2, within the resistance exercise intervention group, there were significant improvements between the pre-test and post-test in depressive symptoms (*p* < 0.05), global cognitive function (*p* < 0.001), visuospatial/executive functions (*p* < 0.05), attention (*p* < 0.05), language (*p* < 0.05), and delayed recall (*p* < 0.05). However, from pre-test to post-test, the intervention group had no significant differences on naming, abstraction, and orientation domains (all *p* ≥ 0.05). Within the control group, there were no significant differences between the pre-test and post-test in depressive symptoms, visuospatial/executive functions, naming, attention, language, abstraction, delayed recall and orientation domains (all *p* ≥ 0.05). However, from pre-test to post-test, the control group had a significant reduction in global cognitive function score (*p* < 0.05).

### 3.4. Comparisons of Pre-Post Improvements between Groups

Table 3 presents the results of the comparisons of the changes from pre-test to post-test in terms of cognitive performance and depressive symptoms between the resistance exercise intervention and control groups. As shown in Table 3, there were significant differences between the intervention and control groups on changes in symptoms of depression (*r* = −0.47, *p* < 0.05), global cognitive function (*r* = −0.87, *p* < 0.001), visuospatial/executive functions (*r* = −0.41, *p* < 0.05), attention (*r* = −0.48, *p* < 0.05), language (*r* = −0.48, *p* < 0.05), and orientation (*r* = −0.41, *p* < 0.05). However, there were no significant differences between both groups on changes in naming, abstraction, and delayed recall domains (all *p* ≥ 0.05).

For sample size estimation in future trials, based on the effect sizes calculated from this pilot study, 23 participants per group would be needed to reach 80% power at the significance level of 0.05 to detect a significant between-group difference in global cognitive function. For detecting a significant between-group difference in depressive symptoms, 76 participants per group would be required to reach 80% power at the significance level of 0.05.

## 4. Discussion

Although exercise interventions have been shown to improve depression and cognition, little is known about the impact of resistance exercise in cognitive function and symptoms of depression in older Chinese Americans. The current study examined the effect of a 12-week resistance exercise intervention on depressive symptoms and cognitive performance among community-dwelling older Chinese Americans. The results showed that the resistance exercise intervention group had significant improvements in depression and global cognitive function compared to the control group. Also, older adults that participated in the resistance exercise program had greater improvements in visuospatial/executive functions, attention, language, and orientation domains than their control group counterparts. However, there were no significant differences between both groups on changes in naming, abstraction, and delayed recall domains.

The existing literature suggests that physical activity interventions were effective in alleviating depressive symptoms [28,29,30]. The findings of this study support previous studies reporting the benefits of resistance exercise on improving depression [31,32,33,57]. Ahmed examined the effect of home-based progressive resistance exercise on depression in older adults [32]. The results indicated that depressive symptoms were reduced significantly in the resistance exercise group. Chen et al. assessed the impact of a 15-month resistance band exercise intervention on depression among older adults with dementia [34]. The finding from this study suggested that the resistance exercise group had better improvements for depression compared to the control group. Singh et al. tested the antidepressant effect of resistance exercise in older adults with major or minor depression [35]. The result showed that older adults that participated in a high-intensity resistance exercise program had better antidepressant effects than their counterparts. However, Chin et al. found that resistance exercise did not significantly relieve depressive symptoms among older persons living in long-term care institutions [36]. Factors related to the levels of depression may include residential settings, health status, gender, income, education, social environment, etc. [23,57]. Future studies on the antidepressant effect of resistance exercise should consider the risk factors of depression when developing personalized interventions among community-dwelling older adults.

The result of the present study showed that resistance exercise significantly improved global cognitive function, which is aligned with previous studies [7,13,19,58]. The findings from this study also showed that the resistance exercise group had greater improvements in visuospatial/executive functions, attention, language, and orientation domains than the control group. Cassilhas et al. evaluated the effects of 24 weeks of resistance exercise with two different intensities on cognition in older adults [7]. The findings have demonstrated that the high-intensity resistance exercise group had better cognitive performance on attention, short-term memory, central executive/digit span, and long-term episodic memory when compared to the control group. The results also revealed that the moderate-intensity resistance exercise group had better improvements in short-term memory, central executive/digit span, and long-term episodic memory than the control group. In addition, Lü et al. examined the impact of a 12-week dumbbell training in community-dwelling older adults with mild cognitive impairment [19]. The result showed that compared to the control group, the dumbbell training intervention group had better improvements on global cognitive function. However, there were no significant differences in improving executive function, immediate memory, and attention between the two groups. Previous studies have mainly focused on the effect of physical activity interventions on global cognitive function. Few studies have tested the impact of exercise programs on specific cognitive domains. The effects of resistance exercise on specific cognitive domains remain unclear. More studies are needed to examine the association of resistance training with specific cognitive domains in older adults.

The findings of this study demonstrated that resistance exercise alleviated depressive symptoms and enhanced cognitive performance in community-dwelling older Chinese Americans. Previous studies have suggested the potential mechanisms for interpreting the beneficial effects of resistance exercise interventions on depression and cognition [8,59]. First, existing literature revealed that cognitive function decline was not only due to volume and density loss in certain brain areas, but also due to cardiovascular risk factors [8,57,59]. Cardiovascular risk factors can cause hemodynamic blood flow changes and induce cerebral hypoperfusion, which decreases energy substrate delivery [60,61]. Cognitive functions, such as memory, executive function, verbal fluency, and psychomotor speed, could be affected by cerebral hypoperfusion as well [57,60,61,62]. However, the brain blood flow increased during exercise, thus improving cognitive performance [61,63]. Second, the hippocampus is significantly associated with cognitive function and emotional regulation [57]. Hence, resistance exercise might improve cognition and depression through increasing hippocampus volumes and enhancing hippocampus function [57,64,65]. Third, low levels of insulin-like growth factor-1 (IGF-1) serum concentrations are related to poor cognition in the elderly [7,66]. Resistance exercise might increase IGF-1 serum concentrations, thus enhancing cognitive health [7,67,68]. Lastly, studies have shown that the antidepressant effect of resistance exercise is associated with the changes in monoamine transmitters and neuroimmunological indicators [13,57]. It is possible that resistance training may reduce depressive symptoms through optimizing the levels of C-reactive protein, 5-hydroxytryptamine, cortisol, and norepinephrine [13,57,69]. 

Previous studies did not provide clear recommendations about the most adequate doses of resistance exercise for greatest benefits in improving depression and cognitive performance in community-dwelling older adults [70]. The current study conducted a 12-week progressive resistance exercise intervention (50 min per session, two times weekly), which significantly improved depression and cognitive health in community-dwelling older Chinese Americans. Cassilhas et al. tested the effects of 24 weeks of moderate-intensity and high-intensity resistance exercise interventions (1 hour per session, three sessions per week for each intervention group) on cognitive functioning in the elderly [7]. The findings indicated that both resistance exercise groups had better cognitive performance than the control group. However, there was no significant difference on cognition between the two resistance exercise groups, which suggests that moderate-intensity resistance exercise was as effective as high-intensity resistance exercise in improving cognition in older adults. Notably, previous research showed that light-intensity physical activity had positive effects on cognition [71,72], which implies that exercise interventions can be effective in improving or maintaining cognitive functioning even with light intensity. 

Northey et al. suggested that at least moderate-intensity exercise with a duration of 45–60 min per session was beneficial to cognitive performance [58]. However, Ahn et al. found that a duration of 30–40 min per session was the most effective on cognitive function improvements in older adults with mild cognitive impairment [13]. Similarly, Gordon et al. reported that a shorter duration of resistance exercise (<45 min per session) had greater antidepressant effects than a longer duration (≥45 min per session) [33]. This suggests that a longer duration of resistance exercise does not predict stronger effects on reductions in depressive symptoms. Overall, the dose–response relationships of resistance exercise with depression and cognition are still unclear. To determine the optimal dose of resistance exercise for maximal benefits in older adults, future research with randomized controlled trials should investigate the dose–response effects of resistance exercise on depressive symptoms and cognitive functioning.

As for the intervention effects in cognition and depression by exercise type, existing literature suggested that resistance exercise, aerobic exercise, multicomponent exercise training, and Tai Chi had beneficial effects on cognitive performance and/or depressive symptoms [13,14,31,58,73,74]. However, Ahn et al. reported that multicomponent exercise (aerobic exercise + resistance exercise) and neuromotor exercise (e.g., Tai Chi, Qigong) did not significantly improve global cognitive function among older adults with mild cognitive impairment [13]. Consistent with the findings of Ahn et al.’s study, Yoon et al. found that there were no significant differences in the changes in cognitive function between the RI group (resistance exercise + interval training), the RA group (resistance exercise + aerobic exercise), and the control group, indicating that multicomponent exercises did not significantly enhance cognition in comparison to the control group among old women [13,75]. In addition, the results from Ahn et al.’s study revealed that aerobic exercise had superior effects in improving cognitive performance compared to resistance exercise [13]. Conversely, Wang et al. found that resistance exercise was the most effective in enhancing cognitive function [14]. Moreover, the findings of Miller et al.’s study showed that compared to control conditions, mind–body exercise had the greatest effects in mitigating depression, followed by aerobic exercise and resistance training [31]. Overall, compared to other exercise interventions, whether resistance exercise is the most effective in improving depression and cognition remains uncertain. Future research examining the effects of resistance exercise in comparison to different exercise modes on cognitive functioning and depressive symptoms is recommended. 

There are several potential limitations in this study. First, the sample size in the current study was small. Also, in this pilot randomized controlled trial, the groups were not matched on MoCA and GDS scores at baseline. In addition, in the intervention group (*n* = 14), 10 participants had improvements in depressive symptoms, but 4 participants did not have improvements in depressive symptoms from pre-test to post-test. Future research to examine factors influencing the resistance exercise intervention effect on depression is recommended. Additionally, this study did not examine the long-term effects of the intervention. In the future, large-scale trials with a longer follow-up to explore the effectiveness of resistance exercise in terms of cognition and depression are necessary. Moreover, outcome assessors were not blinded to the group of the subjects. Furthermore, in this pilot study, the group-based resistance exercise intervention did not compare with home-based/web-based interventions or other physical activity programs. It is not clear if this resistance exercise intervention has superior effects in improving depression and cognition compared to other exercise interventions. Finally, as the participants were community-dwelling older Chinese Americans, it is unknown if the findings of the present study can be extrapolated to other racially and ethnically older populations, or older adults who live in hospitals or long-term care facilities.

## 5. Conclusions

The results of the current study showed that older adults who participated in a resistance exercise group had greater improvements in depression and cognition compared to those in the control group. Consistent with previous studies, this study confirms that resistance exercise significantly enhances cognitive performance and reduces depressive symptoms among older adults. In the future, larger randomized controlled trials with long-term follow-up should be conducted to investigate the optimal exercise prescription for promoting the greatest improvements on depression and cognitive health in older adults.

## Figures and Tables

**Table 1:** Characteristics of the sample at baseline.

Variables	Total (*n* = 28)	Intervention Group (*n* = 14)	Control Group (*n* = 14)	*p*-Value
Age (years; mean ± SD)	77.68 ± 5.11	76.79 ± 4.79	78.57 ± 5.44	0.365 ^a^
Gender, *n* (%)				1.000
Male	6 (21.4)	3 (21.4)	3 (21.4)	
Female	22 (78.6)	11 (78.6)	11 (78.6)	
Education, *n* (%)				0.403
High school or below	20 (71.4)	11 (78.6)	9 (64.3)	
Some college, college graduate, or higher	8 (28.6)	3 (21.4)	5 (35.7)	
Marital status, *n* (%)				0.705
Married	15 (53.6)	7 (50.0)	8 (57.1)	
Unmarried	13 (46.4)	7 (50.0)	6 (42.9)	
Depressive symptoms (GDS score) (mean ± SD)	4.36 ± 3.02	5.43 ± 2.98	3.29 ± 2.75	0.059 ^a^
Global cognitive function (MoCA total score) (mean ± SD)	19.07 ± 3.79	17.78 ± 2.75	20.35 ± 4.34	0.073 ^a^
Cognitive domains (MoCA subscores) (median, IQR)				
Visuospatial/Executive	2.00 (1.00)	2.00 (2.00)	3.00 (1.00)	0.488 ^b^
Naming	2.00 (1.00)	2.00 (1.00)	2.00 (1.00)	0.570 ^b^
Attention	5.00 (1.75)	4.00 (2.00)	5.00 (1.00)	0.219 ^b^
Language	1.00 (1.00)	0.50 (1.00)	1.00 (1.00)	0.403 ^b^
Abstraction	2.00(1.00)	2.00 (2.00)	2.00 (2.00)	0.895 ^b^
Delayed Recall	3.00 (2.00)	2.00 (3.00)	3.00 (2.00)	0.130 ^a^
Orientation	6.00 (1.00)	5.00 (1.00)	6.00 (1.00)	0.121 ^b^

Note: SD = Standard Deviation; IQR = Interquartile Range; GDS = Geriatric Depression Scale; MoCA = Montreal Cognitive Assessment; ^a^: Independent *t*-test; ^b^: Mann–Whitney U test.

**Table 2:** Pre–post changes on depressive symptoms and cognitive performance within the intervention group and the control group.

Variables	Intervention Group (*n* = 14)			Control Group (*n* = 14)		
	Pre-Test	Post-Test	*p*-Value	Pre-Test	Post-Test	*p*-Value
Depressive symptoms (GDS score) (mean ± SD)	5.43 ± 2.98	4.50 ± 3.34	0.031 ^a^ *	3.29 ± 2.75	3.43 ± 2.56	0.699 ^a^
Global cognitive function (MoCA total score) (mean ± SD)	17.78 ± 2.75	20.85 ± 2.87	<0.001 ^a^ ***	20.36 ± 4.34	19.57 ± 4.30	0.010 ^a^ *
Cognitive domains (MoCA subscores) (median, IQR)						
Visuospatial/Executive	2.00 (2.00)	3.00 (0.00)	0.03 ^b^ *	3.00 (1.00)	3.00 (1.00)	1.000 ^b^
Naming	2.00 (1.00)	2.00 (0.00)	0.180 ^b^	2.00 (1.00)	2.00 (0.00)	0.564 ^b^
Attention	4.00 (2.00)	5.00 (1.00)	0.026 ^b^ *	5.00 (1.00)	4.00 (1.25)	0.166 ^b^
Language	0.50 (1.00)	1.00 (1.25)	0.021 ^b^ *	1.00 (1.00)	1.00 (1.00)	0.414 ^b^
Abstraction	2.00 (2.00)	2.00 (1.00)	0.157 ^b^	2.00 (2.00)	2.00 (1.00)	0.317 ^b^
DelayedRecall	2.00 (3.00)	3.00 (2.00)	0.014 ^a^ *	3.00 (2.00)	4.00 (3.00)	0.564 ^b^
Orientation	5.00 (1.00)	5.00 (1.00)	0.317 ^b^	6.00 (1.00)	5.50 (2.00)	0.053 ^b^

Note: SD = Standard Deviation; IQR = Interquartile Range; GDS = Geriatric Depression Scale; MoCA = Montreal Cognitive Assessment; ^a^: Paired *t* test; ^b^: Wilcoxon signed-rank test; *: *p* < 0.05, ***: *p* < 0.001 (two-sided).

**Table 3:** Comparisons of change scores (post-test–pre-test) on depressive symptoms and cognitive performance between the intervention and control groups.

Variables	Intervention Group (*n* = 14)	Control Group (*n* = 14)	*p*-Value ^a^	Effect Size (*r*) ^b^
	Post-Test–Pre-Test Median (IQR)	Post-Test–Pre-Test Median (IQR)		
Depressive symptoms (Change in GDS score)	−1.00 (2.00)	0.00 (1.00)	0.013 *	−0.47
Global cognitive function (Change in MoCA total score)	3.00 (0.25)	−1.00 (2.00)	<0.001 ***	−0.87
Cognitive domains (Change in MoCA subscores)				
Visuospatial/Executive	0.50 (1.25)	0.00 (0.00)	0.029 *	−0.41
Naming	0.00 (1.00)	0.00 (0.00)	0.156	−0.26
Attention	0.50 (2.00)	−0.50 (1.25)	0.011 *	−0.48
Language	1.00 (1.00)	0.00 (0.00)	0.010 *	−0.48
Abstraction	0.00 (0.00)	0.00 (0.25)	0.949	−0.01
Delayed Recall	0.00 (1.00)	0.00 (2.00)	0.219	−0.23
Orientation	0.00 (0.25)	0.00 (1.00)	0.030 *	−0.41

Note: IQR = Interquartile Range; GDS = Geriatric Depression Scale; MoCA = Montreal Cognitive Assessment; ^a^: Mann–Whitney U test; ^b^: *r* = Z/sqrt(N); *: *p* < 0.05, ***: *p* < 0.001 (two-sided).

## Data Availability

Not applicable.
